# Serum thyroglobulin evaluation on LC-MS/MS and immunoassay in TgAb-positive patients with papillary thyroid carcinoma

**DOI:** 10.1530/ETJ-21-0041

**Published:** 2021-12-07

**Authors:** Eijun Nishihara, Yoshitaka Hobo, Akira Miyauchi, Yasuhiro Ito, Miyoko Higuchi, Mitsuyoshi Hirokawa, Mitsuru Ito, Shuji Fukata, Mitsushige Nishikawa, Takashi Akamizu

**Affiliations:** 1 Kuma Hospital, Center for Excellence in Thyroid Care, Kobe, Japan; 2 ASKA Pharmamedical Co., Ltd. Fujisawa, Japan

**Keywords:** serum thyroglobulin, interference with TgAb, LC-MS/MS, structural recurrence

## Abstract

**Objective:**

This study aimed to elucidate disproportionately low serum thyroglobulin (Tg) values in Tg antibody (TgAb)-positive patients with structural recurrence of papillary thyroid carcinoma (PTC) using liquid chromatography-tandem mass spectrometry (LC-MS/MS).

**Design:**

A retrospective study was performed on 176 patients in whom Tg and TgAb levels were measured between 2016 and 2021. Several comprehensive analyses of Tg-LC-MS/MS with an electrochemiluminescence immunoassay for Tg (Tg-ECLIA) were conducted using serum samples.

**Methods:**

TgAb-positive patients who underwent total thyroidectomy with multiple lung metastases due to PTC were evaluated using Tg-LC-MS/MS and Tg-ECLIA. Tg expression in lymph node metastases and metastatic lesions was evaluated by immunohistochemistry and Tg levels of aspiration washouts were also evaluated. Two *in vitro* assays were performed to elucidate TgAb interference.

**Results:**

Tg concentrations of negative TgAb in both assays were similar (R^2^= 0.99; *n*  = 52). Patients with structural recurrence showed higher Tg values with Tg-LC-MS/MS than with Tg-ECLIA. The undetectable proportion was significantly lower with Tg-LC-MS/MS (31.6%, 6/19) than with Tg-ECLIA (68.4%, 13/19; *P*  = 0.023). The spike-recovery rate and Tg concentrations determined by the serum mixture text (*n*  = 29) were significantly reduced to 75.0% (118.3–88.7%) and 81.3% (107.0–87.0%), respectively, with TgAb using Tg-ECLIA (both *P*  < 0.001) confirming assay interference but not using Tg-LC-MS/MS (91.8–92.3%, *P*  = 0.77 and 98.4–100.8%, *P*  = 0.18, respectively).

**Conclusions:**

TgAb had no effect on the Tg-LC-MS/MS assay but yielded 19–25% lower values in Tg-ECLIA. Tg-LC-MS/MS is preferable for monitoring serum Tg levels in TgAb-positive patients, although those with structural recurrence often had disproportionally low Tg values.

## Introduction

Thyroglobulin (Tg) is a thyroid-specific protein, and its serum levels are useful for monitoring patients with differentiated thyroid carcinomas (DTCs) who underwent total thyroidectomy. However, anti-Tg antibodies (TgAb) are present in approximately 25% of patients with DTCs (1, 2, 3). When using currently commercially available immunoassays, which are the most sensitive for measuring Tg, TgAb can mask the epitopes by competing reagent antibodies for Tg. This interference with TgAb may lead to Tg values that are lower than expected or even undetectable. This Tg underestimation is considered the most serious clinical problem for the clinical follow-up of postoperative patients with DTCs.

Liquid chromatography-tandem mass spectrometry (LC-MS/MS) for Tg quantification has emerged as an alternative method that may not be influenced by TgAb. This method is based on specific peptide quantitation following tryptic digestion of all proteins, including TgAb and immunocapture of Tg-specific peptides (4). Among various Tg peptides following cleavage at lysine or arginine with trypsin, two specific peptides, VIFDANAPVAVR and FSPDDSAGASALLR, have been reported to be reliable targets for Tg-LC-MS/MS (5, 6). Although the sensitivity of Tg-LC-MS/MS (approximately 3 ng/mL) was insufficient for clinical use (5), recently improved reagents and instruments have conferred an analytical sensitivity of 4–0.5 ng/mL (7, 8). This improved sensitivity of Tg-LC-MS/MS has been used to evaluate structural recurrence in patients with thyroid cancer and positive TgAb in several clinical trials (6, 9, 10). However, no clear advantage has been proven for Tg-LC-MS/MS over immunoassays because of undetectable Tg values in approximately 40% of patients with distant metastases.

In this study, we improved the immunocapture step of the FSPDDSAGASALLR peptide for Tg-LC-MS/MS to efficiently examine serum Tg values in clinical practice. To clarify the potential impact of Tg measurement on structural recurrence in TgAb-positive patients with papillary thyroid carcinoma (PTC), we compared the Tg values of our Tg-LC-MS/MS with a commercially available electrochemiluminescence immunoassay for Tg (Tg-ECLIA). Furthermore, we elucidated the degree of interference by TgAb in these assay systems using the two *in vitro* tests described below.

## Materials and methods

### Samples and study groups

Serum samples of total of 176 patients were chosen, in whom Tg and TgAb were measured as part of their routine clinical care at Kuma Hospital between 2016 and 2021. Tg and TgAb were measured using the ECLIA method, as described below. Samples were stored at −80°C until analysis for the present study. Serum samples of 158 patients satisfying the following five criteria were consecutively selected as groups A–E (Table 1). Groups A and B were used for comparative measurement of serum Tg levels using Tg-ECLIA and Tg-LC-MS/MS. Group A was comprised of 52 serum samples that were TgAb-negative with low to moderate levels of Tg (<426 ng/mL). Group B was comprised of 19 sera that were TgAb-positive and a varying range of Tg from 19 patients with multiple pulmonary metastases from PTCs treated with total thyroidectomy and radioactive iodine. Samples included in groups C, D, and E were chosen to evaluate the extent of possible interference by TgAb using the following *in vitro* spike-recovery test and serum mixture test (Supplementary Table 1, see section on supplementary materials given at the end of this article). Group C was comprised of 29 serum samples that were TgAb-negative and Tg <0.9 ng/mL from patients who underwent total thyroidectomy for multinodular goiter. Group D was comprised of 29 sera that were TgAb-positive and Tg <0.9 ng/mL from patients who underwent total thyroidectomy for multinodular goiter with Hashimoto thyroiditis. Group E was comprised of 29 sera that were TgAb-negative with high levels of Tg >2000 ng/mL. All sera were 1–2 mL since they were residual sera from routine clinical practice.

**Table 1:** Five categorized groups consisting of 158 serum samples.

Group	Subjects (*n*)	ECLIA	
		TgAb	Tg (ng/mL)
A	Thyroid lobectomy or total thyroidectomy due to PTC (52)	Neg	0.41–426
B	Total thyroidectomy due to PTC with structural recurrence (19)	Pos	<0.08–2.33
C	Total thyroidectomy due to MNG (29)	Neg	<0.9
D	Total thyroidectomy due to MNG with Hashimoto’s thyroiditis (29)	Pos	<0.9
E	MNG (21) Thyroid carcinomas (6) Benign thyroid diseases: Graves’ disease, subacute thyroiditis (2)	Neg	>2000

PTC, papillary thyroid carcinoma; MNG, multinodular goiter.

### Immunoassays of Tg and TgAb

Immunoassays for Tg and TgAb were performed using the ECLIA method, Elecsys Tg II, and Elecsys TgAb (Roche Diagnostics). The lower limit of quantification (LLOQ) for Tg-ECLIA was 0.08 ng/mL, and the cut-off value for TgAb was 28 IU/mL.

### LC-MS/MS analysis procedure

Tg, trypsin, and sodium deoxycholate (DOC) were purchased from Sigma-Aldrich. The internal standard peptide comprising the sequence F*SPDDSAGASALLR (F* [^13^C_9_], mass shift 9 Da) was synthesized by ASKA Pharmamedical Co., Ltd. (Fujisawa, Japan). Dithiothreitol (DTT) and trichloroacetic acid (TCA) were purchased from Nacalai Tesque (Kyoto, Honshu, Japan). Acetic and formic acids were purchased from FUJIFILM Wako Pure Chemical Industries (Osaka, Honshu, Japan). All other reagents used in the experiments were of high grade or equivalent.

Serum (250 µL) was diluted with 250 µL of pure water. Thirty microliters of 5% DOC and 30 µL of 20 mM DTT solution were sequentially added to the sample and incubated at 60°C for 1 h. After adding 10 µL of 4 mg/mL trypsin solution, the mixture was further incubated for 2 h. The enzymatic reaction that digested Tg to its specific peptide (FSP peptide) was quenched with 50 µL of TCA, and an internal standard (IS) was added to it. After centrifugation for 5 min (2150 ***g***, 20°C), the supernatant was purified with polyclonal anti-FSP peptide antibody using MonoSpin ProG (GL Sciences Inc., Tokyo, Japan), which is a spin-type column immobilized with protein G. After purification, the FSP peptide and IS were quantified using LC-MS/MS.

An analysis was performed with heart-cutting 2D-LC-MS/MS using Agilent 1290 Infinity LC (Agilent) for HPLC and API5000 (AB SCIEX) for the MS/MS system. The mobile phase consisted of a gradient elution of 0.1% formic acid solution and acetonitrile for the first separation using ACQUITY UPLC HSS Cyano (1.8 µm, 100 × 2.1 mm id) from Waters (Milford, MA, USA) and a gradient elution of 1 mM acetic acid solution and methanol for the second separation using Triart C18 (1.9 µm, 50×2.1 mm id) from YMC (Kyoto, Japan), with flow rates of 0.5 and 0.45 mL/min, respectively. The column temperature was set to 60°C. The injection volume was 20 µL. Mass transitions monitored in the method were m/z 704.1/587.0 for the FSP peptide and m/z 708.5/586.6 for the IS.

The standard calibration curve was prepared with eight samples at concentrations of 0, 0.4, 1, 4, 20, 40, 200, and 400 ng/mL. PBS was used as a surrogate matrix for standard samples. Peak integration and calculation of concentrations against the standard curve were performed using Analyst version 1.6 (AB SCIEX). The method was fully validated according to the FDA Bioanalytical Method Validation Guide and applied to the analysis of clinical samples. The mean CVs were 4.5% for the intra-assay and 5.7% for the inter-assay. A 0.4 ng/mL LLOQ, the lowest concentration of analyte in a sample that can be quantified reliably, was determined as the analyte signal of the LLOQ sample being at least five times the signal of a blank sample. The precision (%CV) of the concentrations determined at the LLOQ was 10%. The LC-MS/MS assays in this study were performed at ASKA Pharmamedical Co., Ltd. (Fujisawa, Japan).

### *In vitro* tests to evaluate possible interference of the measurements with TgAb

#### Spike-recovery test

To evaluate TgAb interference, 50 µL of standard Tg solution (0.5 μg/mL) was spiked into each of the 200 µL serum samples with Tg <0.9 ng/mL and negative TgAb (group C) and to each of the 200 µL serum samples with Tg <0.9 ng/mL and positive TgAb (group D), to obtain a final concentration of 100 ng/mL for Tg. Each sample was kept at room temperature for 30 min and processed for Tg measurement using Tg-ECLIA and Tg-LC-MS/MS. Tg recovery was calculated as follows: (measured Tg concentration after addition of standard Tg (ng/mL)/100 ng/mL) × 100 (%). The comparative ratio (%) was calculated as the median recovery rate defined as follows: TgAb × 100/median recovery rate without TgAb.

#### Serum mixture test

As an assay to evaluate possible interference by TgAb, each of 200 µL of the 29 serum samples of patients with high Tg concentration and negative TgAb (group E) was randomly mixed with an equal amount of the 29 serum samples with Tg <0.9 ng/mL and negative TgAb (group C). Similar mixtures were also performed for the 29 serum samples with Tg <0.9 ng/mL and positive TgAb (group D). After mild vortexing, the 58 mixed samples were kept at room temperature for 30 min. Serum Tg levels were then measured using Tg-ECLIA and Tg-LC-MS/MS. The change rate of Tg concentration was calculated as follows: (Tg concentration of the mixed group C or D samples (ng/mL) × 2/Tg concentration of a group E sample (ng/mL)) × 100 (%). The comparative ratio (%) was calculated as the median recovery rate defined as follows: TgAb × 100/median recovery rate without TgAb.

#### Assessment of equilibrated Tg with TgAb

To evaluate equilibrated Tg with TgAb dependent on reaction time, both spike-recovery and serum mixture tests were further assessed under three different conditions (at room temperature for 30 min, at 4 °C for 24 h, and at 4 °C for 72 h) in each procedure. Serum samples of 18 patients were additionally used for analysis, including six samples compatible with groups C and D and the other six samples with negative TgAb and moderate Tg levels (around 100 ng/mL) using the ECLIA method.

### Evaluation of Tg production

In TgAb-positive patients with structural recurrence and undetectable Tg levels (Table 2), the following were added:

#### Aspiration washout

Aspiration biopsy of the cervical lymph node metastasis and measurement of Tg in the washout fluid of the needle with saline (0.5 mL) was performed.

#### Tg immunohistochemistry

After surgery, the thyroid and cervical lymph nodes were fixed in 10% neutral buffered formalin, and the specimens were embedded in paraffin. Serial sections (3-µm thick) were cut from each paraffin block.

For six patients with structural recurrence but undetectable Tg values determined by Tg-ECLIA and Tg-LC-MS/MS, immunostaining for human Tg (mouse monoclonal, 1D4, 1:200, Leica Biosystems Newcastle, United Kingdom) was performed using the Leica Bondmax system (Leica Microsystems, Wetzlar, Germany) and a Bond Refine Kit (Leica Microsystems), according to the manufacturer’s instructions.

### Statistical analysis

Linear regression with the least-squares method was used to calculate the regression line equation and correlation coefficient. Differences in percentages between negative and positive TgAb samples in the serum mixture test and spike-recovery test were evaluated using the Mann–Whitney *U* test. Comparisons of the detectable Tg samples between Tg-LC-MS/MS and Tg-ECLIA were performed using the chi-squared test. Statistical significance was set at *P*  < 0.05. Statistical analyses were performed using StatFlex version 6.0 (Artech Co. Ltd., Fukuoka, Japan).

The study protocol was approved by the Ethics Committee of Kuma Hospital (approval number: 20170914-10). Informed consent was obtained from each patient prior to initiation of the study.

## Results

**Figure 1:**
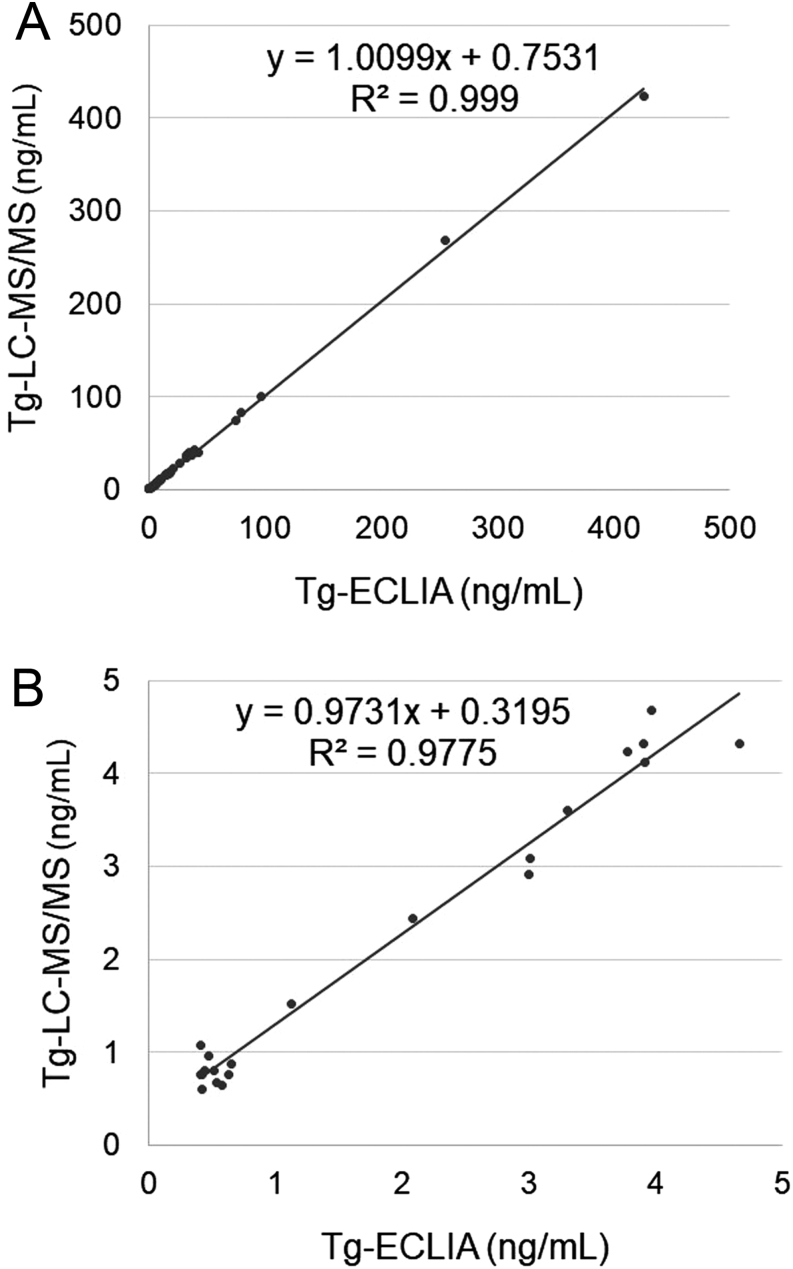
Comparison of serum Tg values with LC-MS/MS and ECLIA measurements in TgAb-negative patients. (A) Total patients (*n*  = 52); (B) subpopulation with <5 ng/mL in the ECLIA method (*n*  = 21).

Figure 1 shows the comparative Tg measurements between LC-MS/MS and ECLIA methods in group A. Tg concentrations in both assays were similar in negative TgAb (R^2^= 0.99; Fig. 1A). A comparison of clinically lower Tg concentrations (<5 ng/mL) also showed a good correlation coefficient and slope bias between both assays (Fig. 1B).

**Figure 2:**
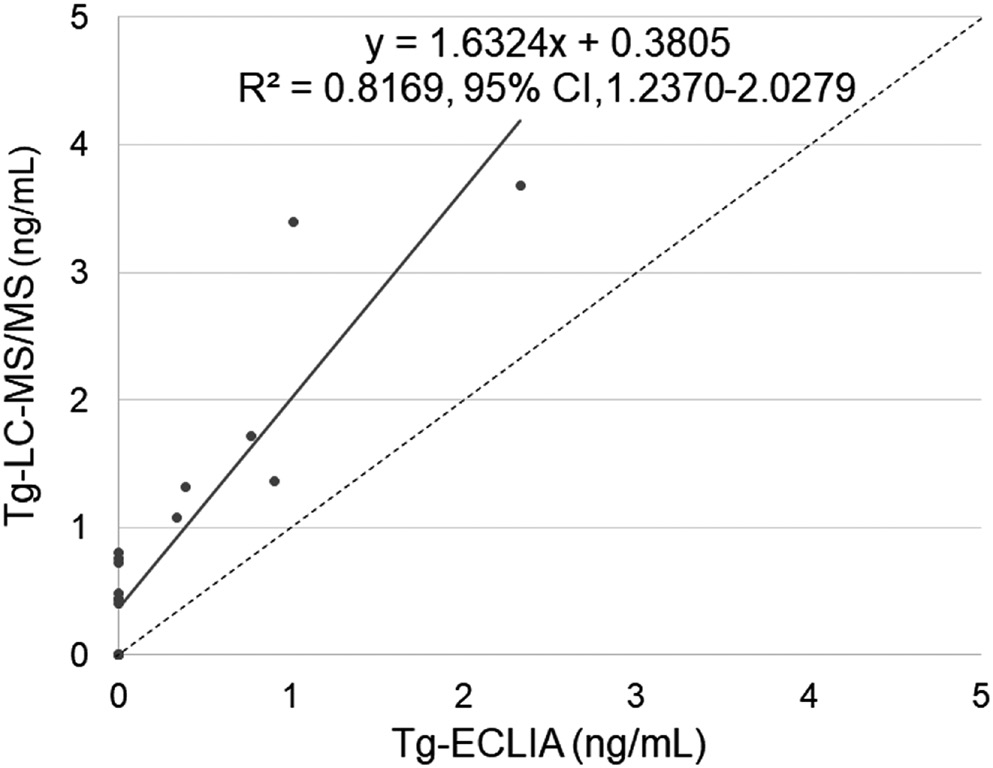
Highly detectable Tg with LC-MS/MS compared with ECLIA in TgAb-positive patients with structural disease of papillary thyroid carcinoma. Serum Tg values in patients (*n*  = 19) with structural disease and positive TgAb using Tg-LC-MS/MS and Tg-ECLIA. The dotted lines indicate equal values between the Tg-LC-MS/MS and Tg-ECLIA.

In the 19 TgAb-positive patients with multiple pulmonary metastases from PTC who had been treated with total thyroidectomy and radioactive iodine (group B), Tg was undetectable in Tg-ECLIA using 13 serum samples. Of these samples, six were undetectable in Tg-LC-MS/MS; however, seven were detectable (Table 2). Although 0.4 ng/mL LLOQ in Tg-LC-MS/MS was higher than that in Tg-ECLIA (0.08 ng/mL), the undetectable percentage (31.6%, 6/19) in Tg-LC-MS/MS was significantly lower than that (68.4%, 13/19) in Tg-ECLIA (*P*  = 0.023). These undetectable Tg values were not dependent on TgAb titer (Table 1). While only six samples showed detectable Tg in both assays, all Tg values obtained were higher with Tg-LC-MS/MS than with Tg-ECLIA (Fig. 2 and Table 2). However, these Tg values were disproportionally low, considering that these patients had structural disease recurrence of PTC. Among group A, five TgAb-negative patients who were treated with total thyroidectomy and radioactive iodine presented with multiple pulmonary metastases from PTCs. Their serum Tg levels were 6.7, 15.7, 16.6, 33.8, and 100.3 ng/mL with Tg-LC-MS/MS.

**Table 2:** Serum Tg values measured with LC-MS/MS and ECLIA methods in TgAb-positive patients with structural papillary thyroid carcinoma.

Patient	TgAb (IU/mL)	Tg (ng/mL)	
		Tg-LC-MS/MS	Tg-ECLIA
1	>4000	3.40	1.01
2	>4000	1.72	0.77
3	>4000	0.80	<0.08
4	>4000	0.44	<0.08
5	>4000	0.40	<0.08
6	>4000	<0.40	<0.08
7	>4000	<0.40	<0.08
8	>4000	<0.40	<0.08
9	2830	0.76	<0.08
10	1805	0.44	<0.08
11	1190	1.36	0.9
12	1013	<0.40	<0.08
13	970.8	0.72	<0.08
14	283.9	1.32	0.39
15	167.8	<0.40	<0.08
16	133.4	1.08	0.34
17	67.9	0.48	<0.08
18	58.8	3.68	2.33
19	56.4	<0.40	<0.08

**Figure 3:**
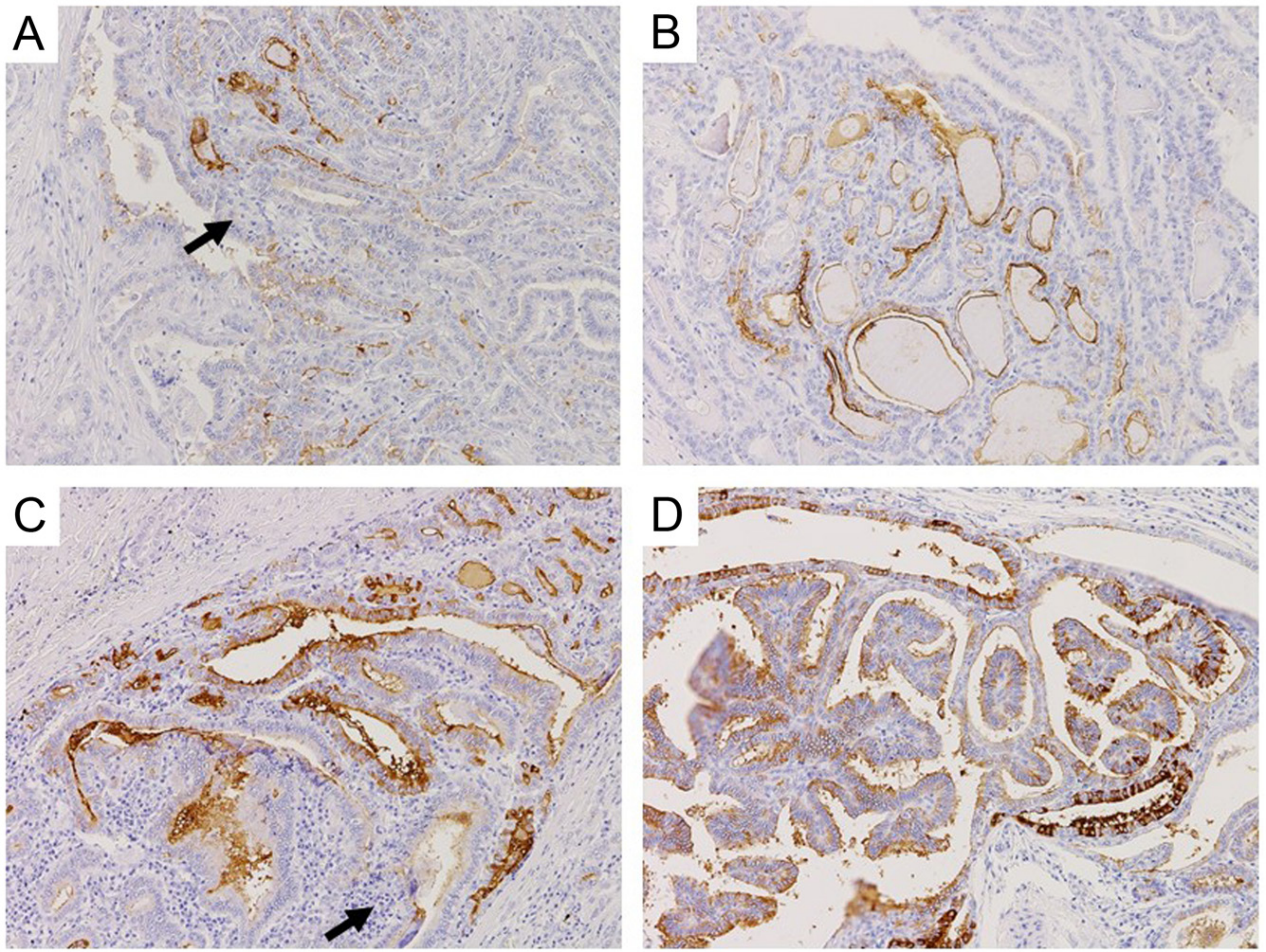
Immunohistochemical analysis of Tg expression in patients with structural disease and positive TgAb. Tg expression was detected at varied levels in conventional histology of PTC in the thyroid (A, C) and lymph node metastasis (B, D). Lymphocytic infiltration in the thyroid is indicated by arrows. A and B, patient 12; C and D, patient 15 in Table 1. Original magnification: ×10 (A–D).

Immunohistochemical analysis of cervical lymph node metastases with conventional histology of PTC in six patients who had structural recurrence but undetectable Tg values in both assays showed that all samples were positive for Tg protein similar to the primary tumors in the thyroid (Fig. 3). In one patient (no. 17 in Table 2), who has developed cervical lymph node metastasis of PTC, Tg levels in the washout fluid after fine needle aspiration were 75.6 ng/mL with Tg-ECLIA and 78.7 ng/mL with Tg-LC-MS/MS, respectively, demonstrating production of Tg in the metastatic lesion.

**Figure 4:**
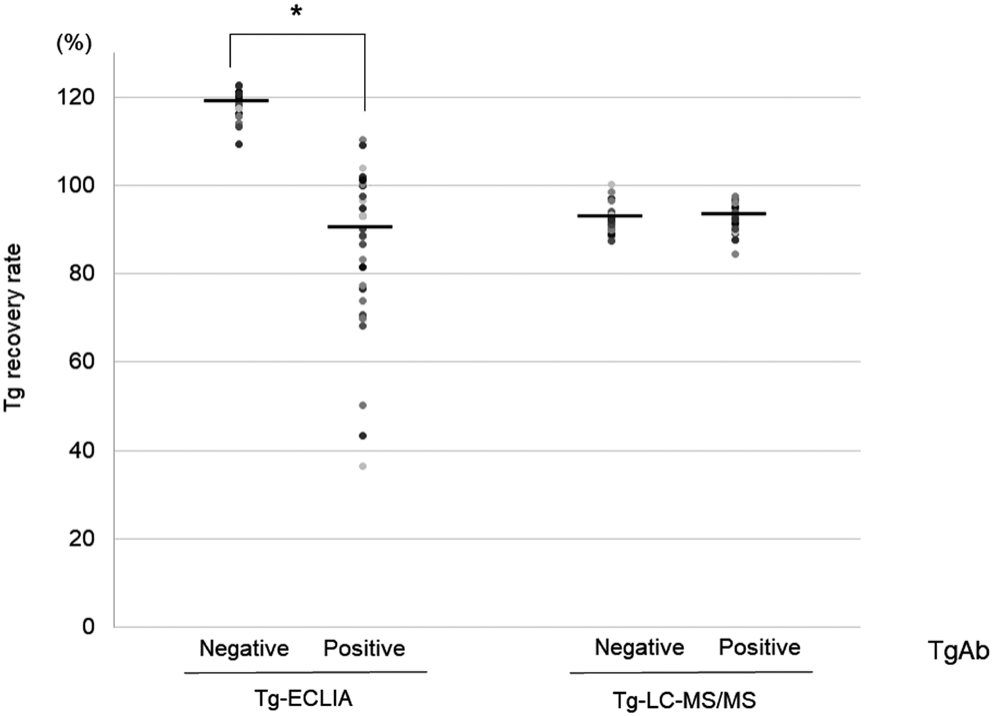
Spike-recovery test showing interference of TgAb with Tg-ECLIA but not with Tg-LC-MS/MS. A standard Tg solution (0.5 μg/mL) was spiked into each serum sample with Tg <0.9 ng/mL and negative TgAb (*n*  = 29) and to each serum sample with Tg <0.9 ng/mL and positive TgAb (*n*  = 29). Tg recovery was calculated as (measured Tg concentration after addition of standard Tg (ng/mL)/100 ng/mL) × 100 (%). Bars indicate the median values. *P< 0.001; negative TgAb vs positive TgAb.

To clarify the possible assay interference by TgAb, we performed two *in vitro* studies. In the spike-recovery tests, the recovery rates of TgAb-positive samples in the Tg-ECLIA assay were diverse and significantly reduced to 75.0% compared with those of TgAb-negative samples (118.3–88.7%, *P*  < 0.001; Fig. 4). In contrast, the recovery rates in the Tg-LC-MS/MS assay were not significantly different, irrespective of the presence or absence of TgAb (*P*  = 0.77), with a comparative ratio of 100.5% (Fig. 4).

**Figure 5:**
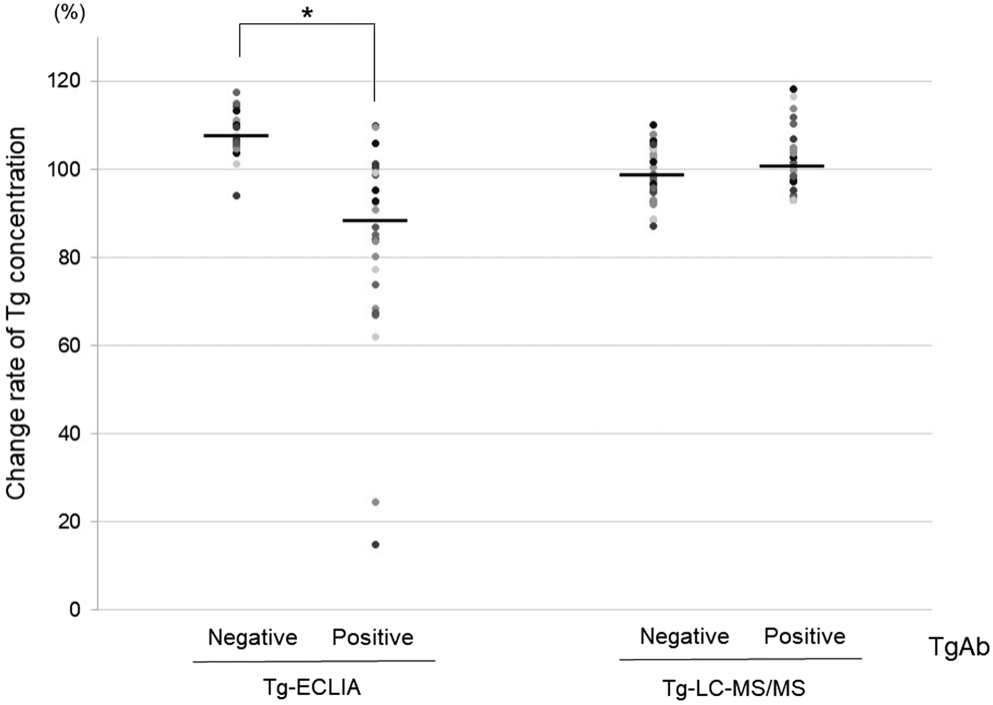
Serum mixture test showing interference of TgAb with Tg-ECLIA but not with Tg-LC-MS/MS. Serum samples of patients with high Tg concentration and negative TgAb (*n*  = 29) were mixed with equal amounts of serum samples (Tg <0.9 ng/mL) and negative TgAb (*n*  = 29). Similar mixtures were also performed for serum samples with Tg <0.9 ng/mL and positive TgAb (*n*  = 29). The change rate of Tg concentration was calculated as (Tg concentration of the mixed samples (ng/mL) × 2/Tg concentration of pre-mixture (ng/mL)) × 100 (%). Bars indicate the median values. *P< 0.001; negative TgAb vs positive TgAb.

In the serum mixture tests, target serum samples with high Tg and negative TgAb were mixed with TgAb-positive sera. The change rates of Tg in the Tg-ECLIA assay were diverse and significantly reduced to 81.3% compared to those with TgAb-negative mixtures (107.0–87.0%, *P*  < 0.001; Fig. 5). In contrast, these mixtures in the Tg-LC-MS/MS assay showed similar rates (*P*  = 0.18), with a comparative ratio of 102.4% (Fig. 5). These *in vitro* studies showed that the extent of interference in Tg-ECLIA by TgAb resulted in a mild decrease in Tg values by 19–25% and that Tg-LC-MS/MS measurement was unaffected by TgAb. Equilibration of Tg with TgAb for a longer time had little effect on both spike-recovery and serum mixture tests (Supplementary Tables 2 and 3).

## Discussion

In this study, we found that patients with structural recurrence of PTCs and positive TgAb had undetectable Tg values of 68.4% with Tg-ECLIA and 31.6% with Tg-LC-MS/MS. Moreover, all detectable Tg values in the Tg-LC-MS/MS assay were disproportionately low for patients with pulmonary metastases of PTC. These data are very similar to those of three previous reports showing undetectable Tg in approximately 40% of patients with structural disease of DTCs and low Tg values on LC-MS/MS measurement (6, 9, 10). Higher levels of TgAb titers were not mainly caused by the degree of Tg underestimation (Table 2) (6).

We conducted *in vitro* assays to evaluate the extent of TgAb interference in Tg-LC-MS/MS and Tg-ECLIA assays. The interference in TgAb is determined by the spike-recovery test, in which a recovery of <80% is considered a positive result (11). In our study, the recovery was 75% in the Tg-ECLIA assay but almost 100% in the Tg-LC-MS/MS assay (Fig. 4). The measured recovery is dependent on the protocol used, including the exogenous Tg concentration and Tg source (12). Therefore, we performed serum mixture tests using samples of various concentrations of native Tg. With TgAb, the Tg concentrations were significantly reduced in Tg-ECLIA but not in Tg-LC-MS/MS (Fig. 5), which supports the spike-recovery test. Our *in vitro* tests showed that the presence of TgAb rendered Tg values 19–25% lower than the expected value in the Tg-ECLIA assay but not in the Tg-LC-MS/MS assay, confirming TgAb interference in the immunoassay. However, the extent of the interference was too small to explain undetectable or low serum Tg values in patients with structural recurrence.

We confirmed the existence of Tg protein in metastatic lesions using immunohistochemistry and Tg measurement of the washout fluid, even in patients with undetectable serum Tg values. Although evaluation of washout fluid from lymph node metastasis is available for only one patient, Tg levels were almost similar in Tg-ECLIA and Tg-LC-MS/MS. Therefore, it is unlikely that serum Tg levels are reduced because of poorly differentiated changes in PTC.

In addition to de-differentiation, there are several possibilities for serum Tg to be much lower than expected in both assays. One is that Tg may be altered by somatic mutations in the* TG* gene in the tumors. The other is that multimerization of Tg at very high concentrations reduces soluble capability and is not recognized by antibodies directed against specific Tg epitopes (13). Otherwise, disproportionally low levels of Tg may reflect low serum Tg concentrations due to the removal of Tg from the bloodstream by some mechanism. An accelerated metabolic clearance of Tg-TgAb complexes due to its greater immune elimination from the blood stream was demonstrated in an animal experiment on autoimmune thyroiditis (14). Similar clinical evidence also suggests that this phenomenon plays a role in humans, wherein serum Tg clearance is parallel with the temporal elevation of specific TgAb immunoglobulin subclasses in patients with Graves’ disease after radioiodine treatment (15, 16). However, the detailed mechanism of Tg clearance remains unclear.

The comparative features of Tg-LC-MS/MS and Tg-ECLIA are summarized in Table 3. In this study, we confirmed that Tg-LC-MS/MS is a valuable alternative method for evaluating Tg values without TgAb interference. In our assay, the introduction of a polyclonal antibody, with the spin-type column immobilized with protein G, resulted in a shorter purification time (from more than 8 h to only 30 min), with an adequate recovery rate (approximately 80%) before liquid chromatography, but it still requires complex and manual workflows, which is a limitation for routine application in clinical practice.

**Table 3:** Comparison of measurements by Tg-LC-MS/MS and Tg-ECLIA.

	Tg-LC-MS/MS	Tg-ECLIA
Time of measurement	1–2 days	20 min
Relative assay cost	6	1
Lower limit of quantification	0.4 ng/mL	0.08 ng/mL
%CV of interassay	4.5%	<10%
Serum Tg values without TgAb	Nearly equivalent to each other	
Interference with TgAb *in vitro*	No	Yes (19–25% decrease)
Serum Tg values with TgAb	Very low	Extremely low

In conclusion, Tg-LC-MS/MS assay was not affected by TgAb, while Tg-ECLIA showed 19–25% lower values in the presence of TgAb. Tg-LC-MS/MS is preferable for monitoring serum Tg levels in TgAb-positive patients. However, TgAb-positive patients with structural recurrence often had disproportionally low Tg values even with the LC-MS/MS assay, indicating some mechanism of removal of Tg *in vivo*, such as elimination of the Tg-TgAb immune complex.

## Supplementary Material

Supplementary Table 1. Serum samples using the spike-recovery and serum mixture tests in vitro

Supplementary Table 2. Evaluation of effect of reaction time on spike-recovery test (n=6)

Supplementary Table 3. Evaluation of effect of reaction time on serum mixture test (n=6)

## Declaration of interest

The authors declare that there is no conflict of interest that could be perceived as prejudicing the impartiality of the research reported.

## Funding

This study did not receive any specific grant from any funding agency in the public, commercial, or not-for-profit sectors.

## Statement of ethics

All participants provided informed consent to participate in the study. The study protocol was reviewed and approved by the Ethics Committee of Kuma Hospital (No. 20170914-10) and conducted in accordance with the principles of the Declaration of Helsinki.

## Author contribution statement

E N and A M designed the study. E N, Y I, and Y H extracted the data and performed the statistical analyses. E N and A M wrote the manuscript. Y H conducted the Tg measurements using Tg-LC-MS/MS. K H and M H performed histological and immunochemical analyses. Y I, M I, S F, M N, and T A contributed to patient care and data acquisition. All the authors discussed the results and approved the final manuscript.
