# Supplementary material for: Serum thyroglobulin evaluation on LC-MS/MS and immunoassay in TgAb-positive patients with papillary thyroid carcinoma
Source: Eur Thyroid J. 2021 Dec 7;11(1):e210041. doi: 10.1530/ETJ-21-0041 (PMC9142804; doi:10.1530/ETJ-21-0041)
Supplement: Supplementary Table 1. Serum samples using the spike-recovery and serum mixture tests in vitro [file supplementary_table_1.pdf]

Supplementary Table 1. Serum samples using the spike-recovery and serum mixture tests *in vitro*

Group C		Group D		Group E	
TgAb (IU/mL)	Tg (ng/mL)	TgAb (IU/mL)	Tg (ng/mL)	TgAb (IU/mL)	Tg (ng/mL)
<28	0.1	55.6	<0.08	<28	2190
<28	0.1	67.1	<0.08	<28	2231
<28	0.12	70.7	<0.08	<28	2234
<28	0.13	79.4	<0.08	<28	2238
<28	0.14	81.4	<0.08	<28	2248
<28	0.15	86.6	<0.08	<28	2271
<28	0.15	99.5	<0.08	<28	2285
<28	0.16	107.5	<0.08	<28	2305
<28	0.19	120.8	<0.08	<28	2308
<28	0.21	121.5	<0.08	<28	2343
<28	0.21	123.6	0.24	<28	2375
<28	0.22	247.3	<0.08	<28	2427
<28	0.24	291	0.24	<28	2434
<28	0.25	340.7	<0.08	<28	2468
<28	0.25	381.5	<0.08	<28	2471
<28	0.29	457.1	0.28	<28	2515
<28	0.3	537.3	<0.08	<28	2605
<28	0.33	602.6	<0.08	<28	2653
<28	0.33	661.2	0.41	<28	2735
<28	0.35	931.5	<0.08	<28	2800
<28	0.36	937.5	<0.08	<28	2802
<28	0.37	955.4	<0.08	<28	2906
<28	0.38	1064	<0.08	<28	2910
<28	0.48	1533	<0.08	<28	2956
<28	0.56	1853	<0.08	<28	3108
<28	0.59	1980	<0.08	<28	3186
<28	0.66	>4000.0	<0.08	<28	3614
<28	0.69	>4000.0	<0.08	<28	3970
<28	0.87	>4000.0	<0.08	<28	4212
