# Supplementary material for: Serum thyroglobulin evaluation on LC-MS/MS and immunoassay in TgAb-positive patients with papillary thyroid carcinoma
Source: Eur Thyroid J. 2021 Dec 7;11(1):e210041. doi: 10.1530/ETJ-21-0041 (PMC9142804; doi:10.1530/ETJ-21-0041)
Supplement: Supplementary Table 3. Evaluation of effect of reaction time on serum mixture test (n=6) [file supplementary_table_3.pdf]

Supplementary Table 3. Evaluation of effect of reaction time on serum mixture test (n=6)

Method		Change rate (%)					
		RT 30 min		4℃ 24 h		4℃ 72 h	
		TgAb		TgAb		TgAb	
		Neg	Pos	Neg	Pos	Neg	Pos
ECLIA		102.7	94.8	100.7	96.0	101.2	93.3
		105.5	70.7	104.0	71.6	104.0	69.0
		97.0	56.7	101.0	52.8	101.3	49.1
		95.1	72.3	98.4	67.2	96.0	58.6
		99.8	79.9	103.6	65.4	103.1	83.2
		91.3	65.4	96.3	50.3	95.7	55.0
	Mean	**98.6**	**73.3**	**100.7**	**67.2**	**100.2**	**68.0**
LC-MS/MS		107.6	109.2	108.1	105.7	105.8	107.1
		101.8	108.0	102.3	99.9	95.1	94.6
		101.1	108.4	103.4	107.4	103.4	103.1
		102.5	105.3	105.0	104.9	104.7	103.6
		100.3	115.5	103.6	114.3	102.8	113.0
		101.4	113.3	101.2	116.5	103.5	116.8
	Mean	**102.5**	**110.0**	**103.9**	**108.1**	**102.6**	**106.4**
